# No Evidence of Decreased Artemisinin Efficacy in a High-Transmission Malaria Setting in Mali

**DOI:** 10.4269/ajtmh.2012.12-0344

**Published:** 2012-07-01

**Authors:** Caroline L. Ng, David A. Fidock

**Affiliations:** Department of Microbiology and Immunology and Division of Infectious Diseases, Department of Medicine, Columbia University Medical Center, New York, New York

Artemisinin-based combination therapies (ACTs) have been adopted globally as first-line drugs for the treatment of *Plasmodium falciparum* malaria over the past decade.^1^,^2^ These drugs have proven to be highly successful in treating malarial parasites resistant to the former first-line drugs chloroquine (CQ) and sulfadoxine/pyrimethamine (SP). Combined with expanded *Anopheles* vector control measures, including insecticide-treated bednets and indoor residual spraying, ACTs have helped achieve important reductions in malaria mortality and morbidity.^3^ The World Health Organization (WHO) estimates that, for the period of 2006–2011, these prevention efforts saved ∼750,000 lives. The successes observed with these treatment and control measures contributed to the call by the Bill and Melinda Gates Foundation and the WHO to expand these intervention efforts with the ultimate goal of achieving malaria eradication.^4^ One major threat, however, comes from recent reports of emerging resistance to artemisinins, which was first documented in western Cambodia and more recently in northwestern Thailand.^5^–^8^ This region in Southeast Asia is of particular concern because it was the source of CQ and SP resistance that eventually migrated to Africa,^9^ raising concerns that artemisinin resistance may follow the same trajectory. This would be devastating, as Africa is home to the vast majority of malaria cases and deaths,^10^ and we are still several years away from any other drugs being licensed and available to replace artemisinins should they fail.^11^

Resistance to artemisinin is unlike any other resistance seen to date, and it is inferred by observations of delayed parasite clearance after treatment. An early report from Cambodia used the median parasite clearance time (defined as the time to parasite-negative blood smears post-initiation of treatment), which was found to be substantially slower in Pailin, western Cambodia, compared with Wang Pha, Thailand (84 hours versus 48 hours, respectively).^6^ More recently, researchers have adopted the parasite clearance rate, defined as the time required for the parasitemia (i.e., the percentage of parasitized red blood cells) to fall by one-half during the log-linear phase of parasite clearance (termed the *t*_1/2_*P* or slope half-life).^12^ Importantly, delayed clearance does not translate into frank clinical treatment failure, although it has been associated with an increased risk of late parasite recrudescence.^13^ An increasing prevalence of patients that remain parasitemic (as evidenced by microscopy) on day 3 post-initiation of treatment is also taken as evidence of emerging resistance.^13^ Recent evidence from a large multicenter, longitudinal study in Thailand suggests that decreased parasite susceptibility to artemisinins is emerging near the Thai–Myanmar border.^7^ Although this change might be caused in part by diminishing acquired immunity as malaria control measures reduce local transmission, investigations of parasite genetics associate decreased *in vivo* parasite clearance rates with a heritable component; parasite genome-wide mapping studies are narrowing the search to several candidate loci.^14^

In this issue of the *American Journal of Tropical Medicine and Hygiene*, the work by Maiga and others^15^ reports a detailed clinical study to test whether resistance to artemisinins may be emerging in a high-transmission setting in Africa after several years of ACT use. This finding was achieved by comparing the therapeutic efficacy of the artemisinin-derivative artesunate, used as a monotherapy agent, between clinical trials in 2010–2011 and 2002–2004. The 2010–2011 study enrolled 100 children with uncomplicated *P. falciparum* malaria in a prospective trial; they were treated with a curative 7-day regimen of oral artesunate, and their clinical and parasitological responses were followed. No significant differences between the two study periods were found in terms of the percentage of patients that had cleared 95% of their parasitemias or completely cleared their infections within 24 hours (corresponding to 89–99% and 32–37% of patients, respectively). All patients were blood smear negative by 72 hours and; 100% cure rates were observed 28 days post-treatment. Equal median fever clearance times (∼24 hours) were recorded in both trials. Overall, the data clearly illustrate that artesunate has not become less effective 6 years after the initiation of widespread use of ACTs in Mali.

This study by Maiga and colleagues^15^ is very welcome news, because it shows that widespread ACT use in a high-transmission African setting for several years has not led to any detectable loss of efficacy. The study provides key benchmark data for future periodic surveillance efforts, and it illustrates keen foresight on the part of the clinical team to have implemented an appropriately powered artesunate monotherapy trial to determine the baseline characteristics early in the era of first-line ACT use.^15^ The design of the study by Maiga and others^15^ closely follows new WHO guidelines for assessing parasite clearance dynamics after curative artesunate treatment, and it sets the standard for similar studies needed across Africa.

Why would artemisinin resistance begin to emerge first in Cambodia and not high-transmission settings in Africa? The recent decline in artemisinin efficacy in Cambodia has often been attributed to prior decades of artesunate monotherapy that, due to the short drug half-life, can readily lead to parasite recrudescence and thereby potentially enrich for resistant parasites.^1^,^6^ Other explanations include substandard or counterfeit drugs, less within-host competition between resistant mutant and drug-sensitive parasites, reduced immunity, parasites more commonly being exposed to drug, and possibly, enhanced parasite propensity to acquire resistance compared with high-transmission settings in Africa.^16^ *In vitro* studies to date suggest that resistance is both hard to select for and when obtained, can be unstable.17–20 The expectation that artemisinin resistance inevitably will arise has spurred significant efforts into expanding the antimalarial drug pipeline.^11^ These investments need to be accompanied by support for research into discovering new drugs and drug targets, elucidating drug modes of action, and deciphering mechanisms of resistance. Establishing the concordance between *in vitro* and *in vivo* data is also paramount, and there is a recognized need to implement a coordinated global resistance surveillance network.^21^ For now, ACTs and vector measures remain highly effective, and implementation efforts based on these measures are vital to achieving the ultimate goal of eliminating malaria from endemic regions.
